# Comparison of neutrophil-to-lymphocyte ratio and platelet-to-lymphocyte ratio for the diagnosis of neonatal sepsis: a systematic review and meta-analysis

**DOI:** 10.1186/s12887-023-04094-y

**Published:** 2023-06-30

**Authors:** Lixia Bai, Peihui Gong, Xiaoyun Jia, Xinhua Zhang, Xiuhui Li, Yueqin Zhang, Hao Zhou, Yanan Kang

**Affiliations:** 1 Department of Shanxi Children’s Hospital (Shanxi Maternal and Child Health Hospital), Medical Services Section, Taiyuan, China; 2 Department of Epidemiology, School of Public Health, Shanxi Medical University, Taiyuan, China; 3 Department of Cadre Health Care Management, Second Hospital of Shanxi Medical University, Taiyuan, China; 4 Department of Shanxi Children’s Hospital (Shanxi Maternal and Child Health Hospital), Neonatal Pediatrics, Taiyuan, China

**Keywords:** Neutrophil-To-Lymphocyte ratio, Platelet-To-Lymphocyte ratio, Neonatal sepsis, Diagnostic test, meta-analysis

## Abstract

**Purpose:**

To compare the performance of Neutrophil-to-Lymphocyte Ratio (NLR) with that of Platelet-to-Lymphocyte Ratio (PLR) in diagnosing neonatal sepsis (NS).

**Methods:**

PubMed and Embase were searched for relevant studies from the inception of the databases to May, 2022. The pooled sensitivity (SEN), specificity (SPE), and area under the receiver operator characteristic curve (AUC) were measured.

**Results:**

Thirteen studies involving 2610 participants were included. The SEN, SPE, and AUC of NLR were 0.76 (95%*CI*: 0.61–0.87), 0.82 (95%*CI*: 0.68–0.91), and 0.86 (95%*CI*: 0.83–0.89), respectively, and those of PLR were 0.82 (95%*CI*: 0.63–0.92), 0.80 (95%*CI*: 0.24–0.98), and 0.87 (95%*CI*: 0.83–0.89), respectively. Significant heterogeneity was observed among the studies. Subgroup analysis and meta-regression showed that types of sepsis (*p* = 0.01 for SEN), gold standard (*p* = 0.03 for SPE), and pre-set threshold (*p*<0.05 for SPE) might be the sources of heterogeneity for NLR, whereas the pre-set threshold (*p*<0.05 for SPE) might be the source of heterogeneity for PLR.

**Conclusions:**

NLR and PLR would be of great accuracy for the diagnosis of NS, and the two indicators have similar diagnostic performance. However, the overall risk of bias was high, and significant heterogeneity was identified among the included studies. The results of this study should be interpreted prudently, and the normal or cut-off values and the type of sepsis should be considered. More prospective studies are needed to further support the clinical application of these findings.

## Introduction

Sepsis refers to systemic inflammatory reaction syndrome caused by microbial infection, the pathogens include bacteria, viruses, fungus, and protozoon. Neonatal sepsis (NS) is a common cause of neonatal death [1–3]. It is of high morbidity especially in newborns, with approximately 3 million cases worldwide and a mortality rate ranging from 11–19% [4]. Sepsis falls into early-onset sepsis (EOS, Septicemia that occurs within 72 h after delivery) and late-onset sepsis (LOS, Septicemia that occurs more than 72 h after delivery) according to the time of onset [5]. The condition of infants with sepsis changes rapidly and the treatment remains intractable, leading to a high mortality rate. Early diagnosis and timely intervention are of great importance for improving the prognosis of NS newborns [6].

NS at an early stage often has atypical symptoms and signs. The current gold standard for NS diagnosis is blood culture, which requires a long culturing time with a low positive rate, making the early diagnosis extremely difficult [7]. There is an urgent need for a rapid biomarker and high specificity to help the early identification of NS before getting a positive blood culture. However, there is no excellent biomarker to be used in predicting NS. Numerous biomarkers have already been investigated for the early detection of sepsis. The classification of these markers includes risk prediction, diagnosis, monitoring, and outcome [8, 9]. Procalcitonin and CD14 are demonstrated to be effective markers, while the costs of detection are often unaffordable for low- and middle-income nations like Brazil [9, 10].

Studies have demonstrated that Neutrophil-To-Lymphocyte Ratio (NLR) and Platelet-To-Lymphocyte Ratio (PLR) could be applied as biomarkers for NS [11–13]. The normal ranges of NLR and PLR do not have been unified, which depends on the age and health status of the neonates. NLR and PLR present to be applicable, feasible, and affordable approaches for rapid diagnosis of NS, and are of great significance for the early diagnosis, treatment, and prevention of NS [14]. However, whether NLR or PLR is a better indicator for the diagnosis of neonatal sepsis and its diagnostic accuracy is still debated.

The aim of this study is to summarize the current evidence to evaluate the diagnostic performance of NLR and PLR for NS, and assess the sensitivity and specificity of NLR and PLR for NS diagnosis, so as to provide a reference for clinical early identification of NS.

## Materials and methods

### Search strategy

PubMed and Embase were searched, from inception to May, 2022, for potentially eligible studies using an algorithm based on combined words. Search items mainly included “Neonatal Sepsis”, “NLR”, “neutrophil to lymphocyte ratio”, “PLR”, and “platelet to lymphocyte ratio”. Taking PubMed for example, the search strategy was designed as follows: (NLR OR neutrophil to lymphocyte ratio OR neutrophil-lymphocyte ratio OR PLR OR platelet to lymphocyte ratio OR platelet-lymphocyte ratio)AND(Neonatal Sepsis OR Neonatal Sepsis OR Sepsis, Neonatal OR Neonatal Late-Onset Sepsis OR Late-Onset Sepsis, Neonatal OR Neonatal Late Onset Sepsis OR Neonatal Late-Onset Sepsis OR Sepsis, Neonatal Late-Onset OR Sepsis, Neonatal Late-Onset OR Neonatal Early-Onset Sepsis OR Early-Onset Sepsis, Neonatal OR Early-Onset Sepsis, Neonatal OR Neonatal Early Onset Sepsis OR Neonatal Early-Onset Sepsis OR Sepsis, Neonatal Early-Onset OR Sepsis, Neonatal Early-Onset). Reference lists of included studies were also searched for potentially eligible studies.

### Inclusion and exclusion criteria

Studies meeting the following criteria were included: (a)Evaluating the diagnostic performance of NLR and PLR for NS; (b)Retrospective study, prospective study, or cross-sectional study. Articles will be excluded for the following reasons:(a)Case-report, literature review, conference summary, abstract unavailable, meta-analysis, letter, and comments;(b)Data unextractable;(c)Study reported and published in non-English.

### Data extraction and quality assessment

Data extraction was conducted by two reviewers independently. Extracted data contained: name of the first author, characteristics of the study (publication date, nationality, study design, and gold standard), characteristics of participants, types of sepsis, samples for test, diagnostic cut-off value, true negative (TN), false negative (FN), true positive (TP), and false positive (FP). Any disagreement was settled via discussion between the reviewers.

Quality assessment was conducted by two reviewers independently using the Quality Assessment of Diagnostic Accuracy Studies (QUADAS-2). Each included study was assessed according to the following domains: patient selection, index test, reference standard, and flow and timing. These domains were assessed according to the risk of bias, and the applicability was graded as “high”, “low”, or “unclear” [15]. Disagreements between the reviewers were settled through discussion.

### Statistical analysis

All data analyses were performed using Stata 15.1 software. Pooled sensitivity (SEN) and specificity (SPE) were analyzed using a bivariate random-effect model. The receiver operator characteristic curve (ROC) was plotted and the area under the curve (AUC) was calculated. For an *I*^*2*^>50%, subgroup analysis and meta-regression were performed to identify the sources of heterogeneity, and the sources were classified based on the following aspects: sample size, nationality, study design, gold standard, types of sepsis, source of participants, and diagnostic cut-off value for sepsis) Deek’s funnel plot was provided to assess the publication bias. A *p* value less than 0.05 indicated significant publication bias.

## Results

### Literature search and study selection

**Fig. 1:**
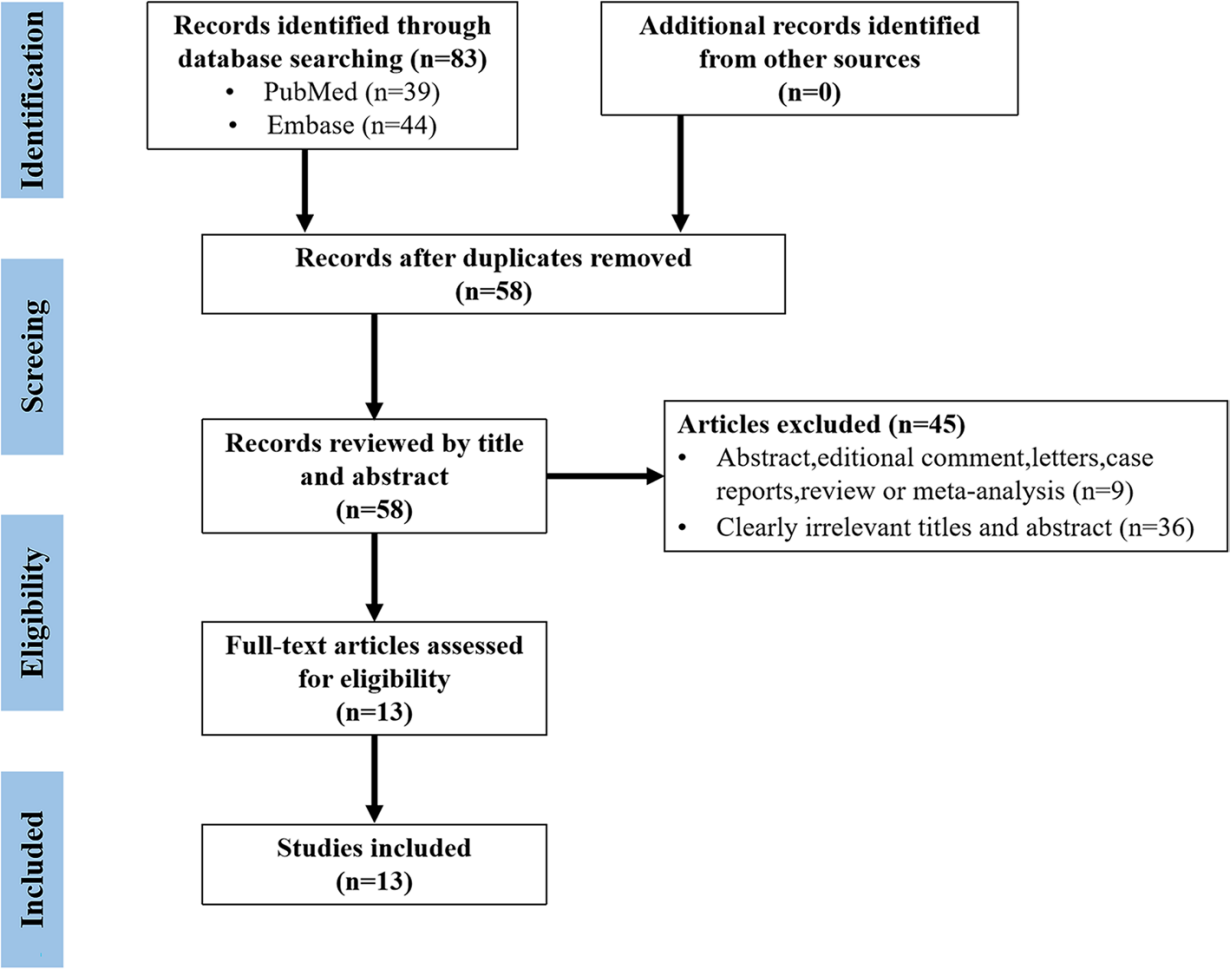
the PRISMA flow diagram of study selection

There were 83 articles retrieved (PubMed: 39, Embase: 44), as shown in Fig. 1, and 25 duplicates were removed. Among the remaining 58 articles, 45 were excluded after browsing titles and abstracts. Finally, 13 studies were included after reading the full texts [13, 14, 16–26].

### Characteristics and quality assessment of included studies

Characteristics of 13 included studies containing 2610 participants (1862 for NLR, 159 for PLR, and 589 for both NLR and PLR) were summarized, as shown in Table 1. Among the 13 included studies, 5 were conducted in Turkey, 3 in China, 2 in Indonesia, 1 in India, 1 in Israel, and 1 in Egypt. As for study design, 6 were retrospective design, 5 were prospective, and 2 were cross-sectional. For the gold standard, 4 studies applied blood culture, 2 applied clinical diagnostic criteria, and 7 adopted both. For types of sepsis, 6 studies reported EOS, 2 reported LOS, and 5 reported both the EOS and LOS.

**Table 1:** The characteristics of the included studies

Author	Year	Country	Study design	Preterm and Term Infants	Gold standard	Type of Sepsis	Specimen	Center	Index	cut-off	N	TP	FP	FN	TN
Kurt, A et al. [16]	2022	Turkey	Retro	Term Infants(100%)	Clinical diagnosis, blood culture	EOS&LOS	Blood	Single	NLR	4.79	134	5	1	52	76
									PLR	37.7	97	16	71	4	6
Zhang,S et al. [17]	2021	China	Pro	Preterm(NA),Term Infants(NA)	Blood culture	EOS	Blood	Single	NLR	3.169	124	57	11	17	39
									PLR	90.846	124	48	10	26	40
Sumitro.K.R et al. [18]	2021	Indonesia	cross-sectional study	Preterm(87.5%),Term Infants(12.5%)	Blood culture	EOS&LOS	Blood	Single	NLR	2.12	104	42	30	10	22
Panda.S.K et al. [13]	2021	India	Retro	Preterm(100%)	Blood culture	EOS&LOS	Blood	Single	NLR	1.7	93	28	28	13	24
Karabulut.B et al. [19]	2021	Turkey	Retro	Term Infants(100%)	Clinical diagnosis	EOS	Blood	Single	NLR	1.42	60	26	5	4	25
Chen.S.J et al. [20]	2021	China	Pro	Preterm(100%),	Clinical diagnosis, blood culture	EOS	Venous cord blood	Single	NLR	NA	427	126	51	26	224
									PLR	50.051	176	73	55	16	32
T. Li, G. Dong et al. [21]	2020	China	Retro	Preterm(NA),Term Infants(NA)	Clinical diagnosis	EOS&LOS	Blood	Single	NLR	1.62	925	376	47	361	141
Goldberg.O et al. [22]	2020	Israel	Retro	Preterm(100%)	Blood culture	LOS	Blood	Single	NLR	1.5	145	42	19	6	78
Wilar.R et al. [23]	2019	Indonesia	cross-sectional study	Preterm(20%),Term Infants(80%)	Clinical diagnosis, blood culture	EOS	Blood	Single	NLR	1.24	120	75	2	15	28
Arcagok, B.C.et al. [14]	2019	Turkey	Retro	Term Infants(100%)	Clinical diagnosis, blood culture	EOS	Blood	Single	PLR	57.70	159	61	2	6	90
Omran, A. et al. [24]	2018	Egypt	Pro	Term Infants(100%)	Clinical diagnosis, blood culture	EOS&LOS	Blood	Single	NLR	2.7	70	28	15	7	20
									PLR	73.10	70	17	19	18	16
Can,E. et al. [25]	2018	Turkey	Pro	Term Infants(100%)	Clinical diagnosis, blood culture	EOS	Blood	Single	NLR	6.76	122	76	0	2	44
									PLR	94.05	122	76	0	2	44
Alkan Ozdemir, S. et al. [26]	2018	Turkey	Pro	Preterm(100%)	Clinical diagnosis, blood culture	LOS	Blood	Single	NLR	1.77	127	38	16	14	59

*Pro* Prospective, *Retro* Retrospective, *EOS* Early onset sepsis, *LOS* Late-onset sepsis, *NA* Not available, *NLR* Neutrophil lymphocyte ratio, *PLR* Platelet lymphocyte ratio, *FN* False negative, *FP* False positive, *TN* True negative, *TP* True positive

**Fig. 2:**
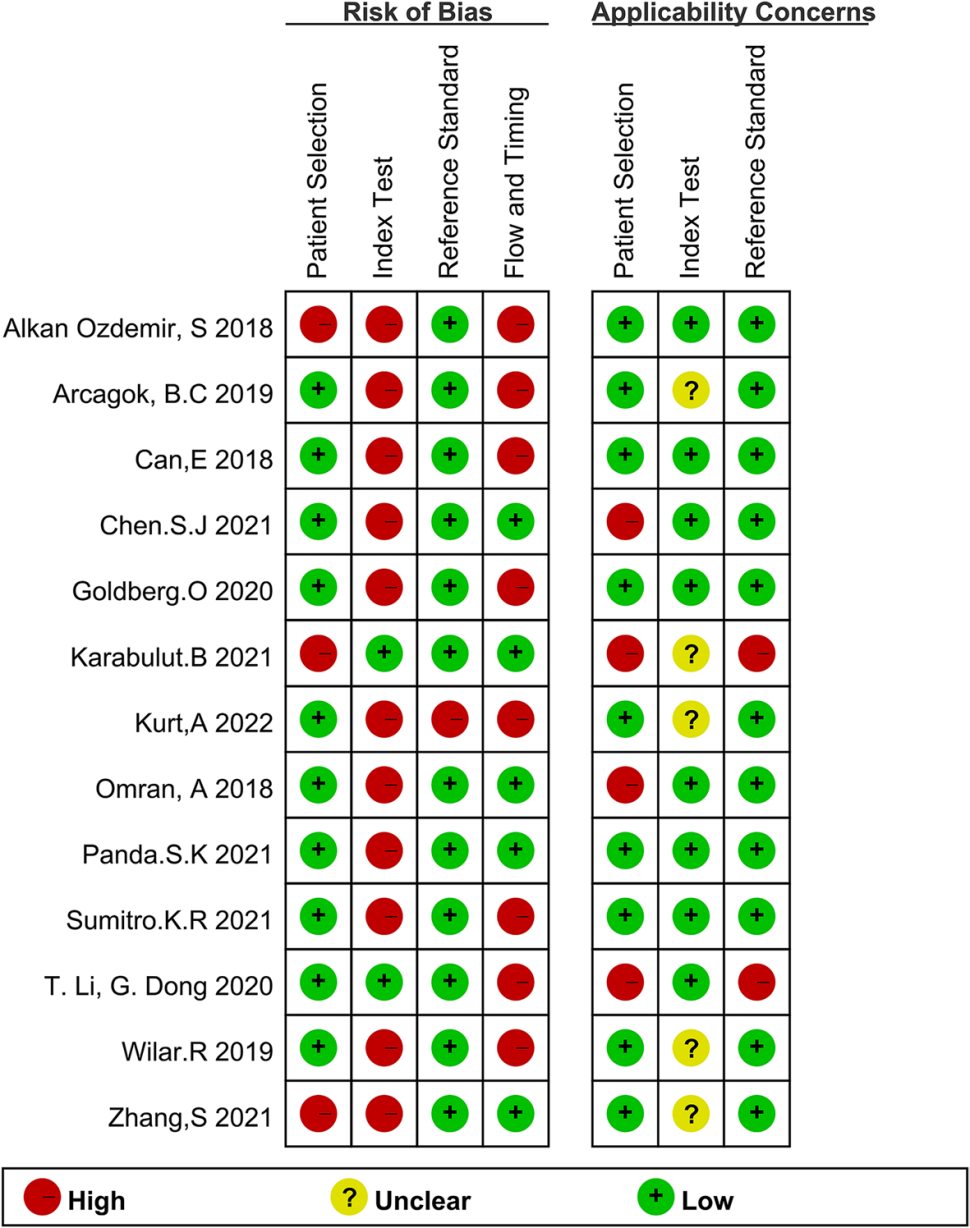
Risk of bias and applicability assessment of included studies

QUADAS-2 was applied to assess the risk of bias and applicability of included studies, as shown in Fig. 2. Quality of included studies was considered to be acceptable. Risk of bias assessment showed that: for patient selection, 3 studies were graded as “high risk” due to non-randomized or discontinuous selection; For index test, 11 studies were graded as “high risk” due to that the cut-off values applied were not pre-determined; For reference standard, 1 study was graded as “high risk” due to application of other criteria for NS diagnosis. For flow and timing, 8 studies were graded as “high risk” due to that part of the participants were excluded from data analyses. Applicability assessment showed that 4 studies were graded as “high applicability” in the selection of NS children in that the participants were concomitant with no other diseases, 5 studies were graded as “unclear applicability” in index test due to that detailed process of diagnostic test was not reported, and 2 studies were graded as “high applicability” in reference standard due to that the gold standard was not applied.

### Diagnostic performance of NLR for NS

The pooled SEN of NLR was 0.76 (95%*CI*: 0.61–0.87), with significant heterogeneity (96.82%). The SPE was 0.82 (95%*CI*: 0.68–0.91), with significant heterogeneity (92.37%). The AUC of NLR was 0.86 (95%*CI*:0.83–0.89).

**Fig. 3:**
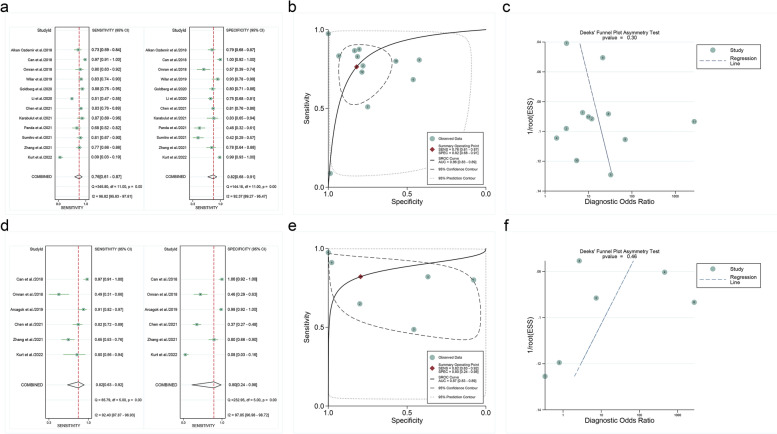
**a** Pooled SEN and SPE of NLR for diagnosing NS, **b** NLR symmetrical summary receiver operator characteristic (SROC) curve for included studies, **c** Deeks’ funnel plot for publication bias, d Pooled SEN and SPE of PLR for diagnosing NS, **e** PLR symmetrical summary receiver operator characteristic (SROC) curve for included studies, f Deeks’ funnel plot for publication bias

Subgroup analysis for EOS showed that the pooled SEN, SPE, and AUC of NLR were 0.87 (95%*CI*: 0.77–0.93), 0.90 (95%*CI*: 0.73–0.97) and 0.94 (95%*CI*: 0.92–0.96), respectively. Detailed results are shown in Fig. 3a and b. The results of subgroup analysis and meta-regression were shown in Table 2.

**Table 2:** Subgroup analysis of diagnostic performance of NLR

Covariate/Subgroup	Studies, n	Sensitivity(95%CI)	*P*-value	Specificity(95%CI)	*P*-value
Number of samples			0.05		0.75
> 130	4	0.58[0.31–0.85]		0.87[0.74-1.00]	
≤ 130	8	0.83[0.72–0.95]		0.78[0.63–0.94]	
Country			0.59		0.60
China	3	0.72[0.43-1.00]		0.79[0.54-1.00]	
Other countries	9	0.78[0.63–0.93]		0.83[0.71–0.96]	
Study design			0.24		0.75
Prospective	5	0.85[0.71–0.99]		0.83[0.68–0.99]	
Retrospective and Clinical	5	0.61[0.35–0.87]		0.83[0.67-0.0.98]	
Gold standard			0.95		0.03
Blood culture	4	0.80[0.58-1.00]		0.64[0.37–0.91]	
Clinic, blood culture	6	0.76[0.56–0.96]		0.91[0.81-1.00]	
Type of Sepsis			0.01		0.11
EOS&LOS	5	0.57[0.33–0.80]		0.72[0.50–0.94]	
EOS	5	0.87[0.76–0.89]		0.89[0.78-1.00]	
Specimen			0.94		0.68
Blood	11	0.76[0.62–0.90]		0.82[0.70–0.94]	
Venous cord blood	1	0.83[0.48-1.00]		0.82[0.44-1.00]	
cut-off			0.54		0.04
> 3	3	0.69[0.35-1.00]		0.96[0.90-1.00]	
≤ 3	8	0.78[0.62–0.94]		0.72[0.57–0.87]	

For all studies on NLR, the median cut off value is 2.12, and then the integer 3 is taken

Subgroup analysis and meta-regression were performed to identify the sources of heterogeneity. Types of sepsis (*p* = 0.01 for SEN), gold standard (*p* = 0.03 for SPE), and the cut-off values (*p<*0.05 for SPE) might be the sources of NLR heterogeneity. Deek’s funnel plot for included studies showed no significant publication bias (Fig. 3c, *p* = 0.30).

### Diagnostic performance of PLR for NS

The pooled SEN of PLR was 0.82 (95%*CI*:0.63–0.92), with significant heterogeneity (92.40%). The SPE was 0.80 (95%*CI*:0.24–0.98), with significant heterogeneity (97.85%). The AUC of PLR was 0.87 (95%*CI*:0.83–0.89).

Subgroup analysis for EOS showed that the pooled SEN, SPE, and AUC of PLR were 0.82 (95%*CI*: 0.63–0.92), 0.80 (95%*CI*: 0.24–0.98) and 0.87 (95%*CI*: 0.83–0.89), respectively. Detailed results are shown in Fig. 3d and e. The results of subgroup analysis and meta-regression were shown in Table 3.

**Table 3:** Subgroup analysis of diagnostic performance of PLR

Covariate/Subgroup	Studies, n	Sensitivity(95%CI)	*P*-value	Specificity(95%CI)	*P*-value
Number of samples			0.49		0.24
>130	2	0.88[0.71-1.00]		0.84[0.24-1.00]	
≤ 130	4	0.78[0.58–0.97]		0.80[0.26-1.00]	
Country			0.47		0.76
China	2	0.74[0.45-1.00]		0.60[-0.36-1.00]	
Other countries	4	0.85[0.71-1.00]		0.87[0.51-1.00]	
Study design			0.65		0.10
Prospective	4	0.80[0.62–0.98]		0.87[0.49-1.00]	
Retrospective and Clinical	2	0.87[0.68-1.00]		0.66[-0.33-1.00]	
Gold standard			0.41		0.31
Blood culture	1	0.65[0.17-1.00]		0.81[-0.09-1.00]	
Clinic, blood culture	5	0.85[0.72–0.98]		0.79[0.34-1.00]	
Type of Sepsis			0.07		0.48
EOS&LOS	1	0.49[0.03–0.94]		0.46[-0.99-1.00]	
EOS	5	0.86[0.75–0.96]		0.85[0.50-1.00]	
Specimen			0.79		0.05
Blood	5	0.81[0.65–0.97]		0.86[0.53-1.00]	
Venous cord blood	1	0.82[0.49-1.00]		0.37[-0.95-1.00]	
cut-off			0.46		0.00
> 90	2	0.88[0.71-1.00]		0.99[0.92-1.00]	
≤ 90	4	0.78[0.59–0.97]		0.53[-0.10-1.00]	

For all studies on PLR, the median cut off value is 81.793, and then the integer 90 is taken

Subgroup analysis and meta-regression were performed to identify the sources of heterogeneity. The cut-off values (*p*<0.05 for SPE) might be the source of PLR heterogeneity. Deek’s funnel plot for included studies showed no significant publication bias (Fig. 3f, *p* = 0.46).

## Discussion

Neutrophil count, lymphocyte count, and platelet count are often utilized as clinical indicators in blood analysis, which entails a quick and accessible laboratory investigation [27]. Indicators of NLR and PLR generated from blood analysis have received interest in the study of inflammation-related disorders in recent years [28]. PLR is increased in the inflammatory response as the microcirculation of the body is altered, the permeability of blood vessels is increased, platelets are activated, and a large number of platelets are aggregated, which in turn aggravates the inflammatory response of the body [29]. NLR is considered to be a more sensitive indicator for microbial infection. It rises rapidly after being infected and is often associated with disease severity [30, 31]. There have been increasing studies demonstrating that NLR and PLR would be of clinical significance for the diagnosis of NS [25, 32, 33], and would be associated with the severity and prognosis of the disease. However, it is still unclear which is the better diagnostic value of NLR or PLR for neonatal sepsis. This study has performed meta-analysis for studies evaluating NLR and PLR for the diagnosis of NS, and has comprehensively assessed the diagnostic value of NLR and PLR, so as to provide a reference for clinical early identification of NS. Subgroup analysis for EOS showed that the pooled SEN, SPE, and AUC of NLR were 0.87 (95%*CI*: 0.77–0.93), 0.90 (95%*CI*: 0.73–0.97) and 0.94 (95%*CI*: 0.92–0.96), respectively. Subgroup analysis for EOS showed that the pooled SEN, SPE, and AUC of PLR were 0.82 (95%*CI*: 0.63–0.92), 0.80 (95%*CI*: 0.24–0.98) and 0.87 (95%*CI*: 0.83–0.89), respectively.

This is the first systematic review and meta-analysis comparing the two indicators for NS diagnosis. Data on the participants in the 13 studies were collected and analyzed, and the results showed that the SEN, SPE, and AUC of NLR were 0.76, 0.82, and 0.86, respectively, and those of PLR were 0.82, 0.82, and 0.87, respectively. Both the two indicators have presented great and similar diagnostic accuracy, which indicates that NLR and PLR are of great accuracy in diagnosing NS, and of remarkable value for clinical screening and definite diagnosis of NS. These two indicators can be considered as reliable biomarkers of early-stage NS.

Deek’s funnel plot showed no significant publication bias existing, while there was significant heterogeneity, among included studies. Subgroup analysis and meta-regression showed that for NLR, types of sepsis might be the source of heterogeneity of SEN, whereas the gold standard and the cut-off values might be that of SPE. As for PLR, the cut-off values might be the source of heterogeneity of SPE, while the source of heterogeneity of SEN could not be identified.

The diagnostic cut-off values for NLR and PLR varied across the included studies, which might induce heterogeneity. Specifically, the cut-off values for NLR ranged from 1.24 to 6.76, while that for PLR ranged from 37.7 to 97.4. The differences in cut-off values could be explained by variations in the study population, the type and severity of sepsis, and the laboratory testing methods used to measure NLR and PLR. Furthermore, the optimal cut-off values for NLR and PLR might depend on the context and the diagnostic performance criteria. Future studies should aim to establish standardized cut-off values for NLR and PLR that can be applied across different populations and settings.

This study also has some limitations: firstly, only 13 studies were included in the meta-analysis, which lead to a small sample size. Secondly, all the included studies are retrospective or cross-sectional, more prospective studies in this area are needed to draw more robust conclusions. Lastly, none of the thresholds of PLR or NLR for the research were settled before the diagnosis, which could lead to an overestimation of their diagnostic value.

## Conclusion

In summary, this meta-analysis has demonstrated that NLR and PLR would be of great sensitivity and specificity in the diagnosis of NS, and can be used as biomarkers for early diagnosis of NS. These two indicators have shown similarly remarkable diagnostic accuracy in the detection of NS, which indicates that NLR and PLR would be accurate and reliable in the diagnosis of NS. Clinical pediatricians can consider using these two laboratory indicators to diagnose NS, with a view to early detection, early diagnosis and early treatment of NS, so as to improve the cure rate of NS and shorten the treatment cycle. However, the overall risk of bias was high, and significant heterogeneity was identified among the included studies. The results of this study should be interpreted prudently, and the normal or cut-off values and the type of sepsis should be considered. More prospective studies are needed to further support the clinical application of these findings.

## Data Availability

The datasets supporting the conclusions of this article are included within the article.
